# Burden of elevated lipoprotein(a) among patients with atherosclerotic cardiovascular disease: Evidence from a systematic literature review and feasibility assessment of meta-analysis

**DOI:** 10.1371/journal.pone.0294250

**Published:** 2023-11-20

**Authors:** Panagiotis Orfanos, Ana Filipa Fonseca, Xingdi Hu, Raju Gautam, Glenn Montgomery, Rachel Studer, Japinder Kaur, Nehul Saxena, Nitin Kaushik

**Affiliations:** 1 Value and Access, Novartis Pharma AG, Basel, Switzerland; 2 Value and Access, Novartis Pharmaceutical Corporation, East Hanover, New Jersey, United States of America; 3 Value and Access, Novartis Healthcare Pvt. Ltd., Hyderabad, India; Centre Hospitalier Sud Francilien, FRANCE

## Abstract

**Background:**

Elevated lipoprotein(a) [Lp(a)] level is an independent genetic risk factor that increases the risk of atherosclerotic cardiovascular disease (ASCVD) by 2–4 fold. We aimed to report the burden of clinically relevant elevated Lp(a) in secondary prevention ASCVD population as the evaluation of such evidence is lacking.

**Methods:**

A systematic literature review (SLR) was conducted using Embase^®^, MEDLINE^®^, and MEDLINE^®^ In-Process databases to identify studies reporting burden of elevated Lp(a) levels from January 1, 2010, to March 28, 2022. Full-text, English-language studies including ≥500 participants with ≥1 Lp(a) assessment were included.

**Results:**

Sixty-one studies reported clinical burden of elevated Lp(a). Of these, 25 observational studies and one clinical trial reported clinical burden of clinically relevant elevated Lp(a) levels. Major clinical outcomes included major adverse cardiovascular event (MACE; n = 20), myocardial infarction (MI; n = 11), revascularization (n = 10), stroke (n = 10), cardiovascular (CV) mortality (n = 9), and all-cause mortality (n = 10). Elevated Lp(a) levels significantly increased the risk of MACE (n = 15) and revascularization (n = 8), while they demonstrated a trend for positive association with remaining CV outcomes. Meta-analysis was not feasible for included studies due to heterogeneity in Lp(a) thresholds, outcome definitions, and patient characteristics.

Three studies reported humanistic burden. Patients with elevated Lp(a) levels had higher odds of manifesting cognitive impairment (odds ratio [OR] [95% confidence interval; CI]: 1.62 [1.11–2.37]) and disability related to stroke (OR [95% CI]:1.46 [1.23–1.72)]) (n = 2). Elevated Lp(a) levels negatively correlated with health-related quality of life (R = −0.166, p = 0.014) (n = 1). A single study reported no association between elevated Lp(a) levels and economic burden.

**Conclusions:**

This SLR demonstrated a significant association of elevated Lp(a) levels with major CV outcomes and increased humanistic burden in secondary prevention ASCVD population. These results reinforce the need to quantify and manage Lp(a) for CV risk reduction and to perform further studies to characterize the economic burden.

## Introduction

Lipoprotein(a) [Lp(a)] is an atherogenic lipoprotein produced in the liver by the covalent assembly of apolipoprotein B-100 with apolipoprotein A [1]. Elevated Lp(a) levels are found in one in five people worldwide and have been associated with an increased risk of cardiovascular disease (CVD) [2–4]. Mendelian randomization studies have established Lp(a) as an independent genetic and causal risk factor for myocardial infarction (MI), ischemic stroke, and coronary artery disease (CAD) in individuals with or without CVD [5–7].

Atherosclerotic cardiovascular disease (ASCVD) is characterized by atherosclerotic plaques resulting from the deposition of lipids in areas of arteries with disturbed nonlaminar blood flow [7]. The 2018 American Cholesterol Clinical Practice Guidelines consider elevated Lp(a) as a risk-enhancing factor for ASCVD [8]. Despite the existing evidence on the association of elevated Lp(a) levels with an increased ASCVD risk, Lp(a) profiling in patients has not been adopted in clinical practice. Lp(a) levels are genetically determined and cannot be significantly modified by diet and exercise [1]. The 2018 American Cholesterol Clinical Practice Guidelines suggest the initiation of statin therapy for patients with elevated Lp(a) [8]. Statins reduce the overall lipid levels, yet they do not have a clinically meaningful impact on Lp(a) levels. Thus, the lack of approved Lp(a)-targeting therapy for lipid management and ASCVD event risk reduction is an unmet need.

Guidelines from the World Health Organization (WHO), International Federation of Clinical Chemistry and Laboratory Medicine (IFCCLM), National Lipid Association (NLA), American College of Cardiology/American Heart Association (ACC/AHA), Canadian Cardiovascular Society (CCS), American Association of Clinical Endocrinologists (AACE), and American College of Endocrinology (ACE) suggest Lp(a) values of ≥50 mg/dL, ≥100 nmol/L, or ≥125 nmol/L as clinically relevant elevated Lp(a) thresholds [9,10].

The European Society of Cardiology (ESC) and European Atherosclerosis Society (EAS) 2019 guidelines recommend Lp(a) measurement to identify patients with very high inherited Lp(a) levels of >180 mg/dL (>430 nmol/L) [9]. In 2022, EAS provided guidance on the increased risk of recurrent ischemic stroke in children with elevated Lp(a) levels defined as >30 mg/dL (>75 nmol/L). EAS also specified Lp(a) ≥300 mg/dL as the maximum Lp(a) concentration in humans [11]. According to Heart UK 2019 recommendations, different Lp(a) serum concentration ranges confer varying degrees of CV risk—minor: 32–90 nmol/L, moderate: 90–200 nmol/L, high: 200–400 nmol/L, and very high >400 nmol/L [9].

Chinese guidelines for the management of dyslipidemia have defined elevated Lp(a) levels conferring CV risk as ≥30 mg/dL [12]. The Australian integrated guidance on patients with familial hypercholesterolemia (FH) with ASCVD has defined elevated Lp(a) levels as ≥150 nmol/L or ≥70 mg/dL [13,14]. In addition, Kamstrup et al. defined the 95^th^ percentile of Lp(a) or ≥90 mg/dL as elevated Lp(a) and a risk factor for aortic valve stenosis and ischemic heart disease in the general population [15].

To the best of our knowledge, no studies have systematically evaluated the disease burden associated with guideline-recommended clinically relevant elevated Lp(a) levels in patients with ASCVD. We performed a systematic literature review (SLR) to understand the clinical, humanistic, and economic burden across clinically relevant Lp(a) levels in the ASCVD population.

## Materials and methods

The SLR was conducted in accordance with the Preferred Reporting Items for Systematic Reviews and Meta-Analyses (PRISMA) guidelines [16]. The PRISMA checklist is provided as S1 Table. Embase^®^, MEDLINE^®^, and MEDLINE^®^ In-Process databases were searched for articles published from January 1, 2010, to March 28, 2022. The MEDLINE^®^ Epub Ahead of Print, In-Process, and Other Non-Indexed Citations were searched on PubMed (March 28, 2022). Searches were limited to English-language articles. A bibliographic search of relevant reviews was performed to identify additional publications. The search strategies along with detailed inclusion and exclusion criteria used to conduct the SLR are provided in S2–S4 Tables.

Briefly, studies were included if they had: (a) patients with established ASCVD, (b) ≥500 participants, and (c) reported clinical, humanistic, or economic burden associated with elevated Lp(a) in the secondary prevention of ASCVD, regardless of the Lp(a) unit (mg/dL vs nmol/L) used for measurement. Due to paucity of evidence for humanistic and economic burdens, the sample size restriction criteria of ≥500 patients was not applied for these reviews. Observational studies, randomized controlled trials (RCTs), post hoc analyses, and meta-analyses were included. Titles and abstracts were screened by two independent reviewers. A third independent reviewer resolved discrepancies by consensus. Full-text publications were screened, and those satisfying the inclusion criteria were selected for data extraction. Multiple publications from the same study were linked and extracted as a single study. Data extraction was performed by one reviewer. Data quality checks were performed by the second reviewer and differences were reconciled by the third reviewer. Observational studies and RCTs were critically appraised on the Newcastle-Ottawa Scale (NOS) [17] and Cochrane Collaboration’s Risk of Bias Tool, respectively [18].

Clinical burden is summarized as clinical outcomes, including composite major adverse cardiovascular event (MACE), cardiovascular (CV) and all-cause mortality, MI, stroke, and revascularization, reported as events percentage, event rates per 100 patient/person-years, and hazard ratio (HR), odds ratios (OR), or relative risk (RR) with 95% confidence intervals (CIs).

Humanistic burden was assessed differently in all the included studies. Cognitive impairment was defined as Montreal Cognitive Assessment (MoCA) scores of ≤22 at 1 year. Cognitive improvement was defined as an MoCA increase of ≥20% or ≥30%. Functional outcome was assessed with the modified Rankin scale (mRS) at 3 months and 1 year after stroke. The mRS scale ranges from 0 to 6. An mRS score of 0 was defined as no residual stroke symptoms, 5 as severe disability, and 6 as death. Increased disability related to stroke was defined as an mRS score of ≥3. Humanistic burden was also assessed using the Short Form Health Survey (SF-36) for health-related quality of life (HRQoL). The HRQoL score included both the physical component summary (PCS) and the mental component summary (MCS), with each comprising four parts. Thus, HRQoL assessed eight items, with each item scoring between 0 (worst) and 100 (best). The weighted standard of the eight items was then converted into the total HRQoL score, with an average of 50±10. The outcomes are reported as patient percentage, events percentage, ORs with 95% CIs, and Spearman’s multiple linear regression correlation coefficient.

Economic burden was reported as median hospitalization cost (in 10,000 yuan). Healthcare resource utilization was measured in terms of length of hospital stay (in days).

The clinical burden has been reported for clinically relevant elevated Lp(a) thresholds, including ≥30/≥50/≥70/≥90/≥180 mg/dL or ≥75/≥90/≥100/≥125/≥150 nmol/L, or super-elevated Lp(a) thresholds, including ≥300 mg/dL or ≥200/≥400/≥430 nmol/L. Due to paucity of evidence for humanistic burden and economic burden, results have been presented for all the elevated Lp(a) thresholds for these reviews.

The outcomes included in the feasibility assessment for network meta-analysis were MACE, CV mortality, all-cause mortality, MI, stroke, and revascularization. The outcomes were identified based on the key clinical outcomes for ASCVD. Heterogeneity in terms of elevated Lp(a) thresholds, references for elevated Lp(a) thresholds, patient populations, study characteristics, patient characteristics, risk factors, comorbidities, and outcomes were assessed.

## Results

### Study characteristics

**Fig 1:**
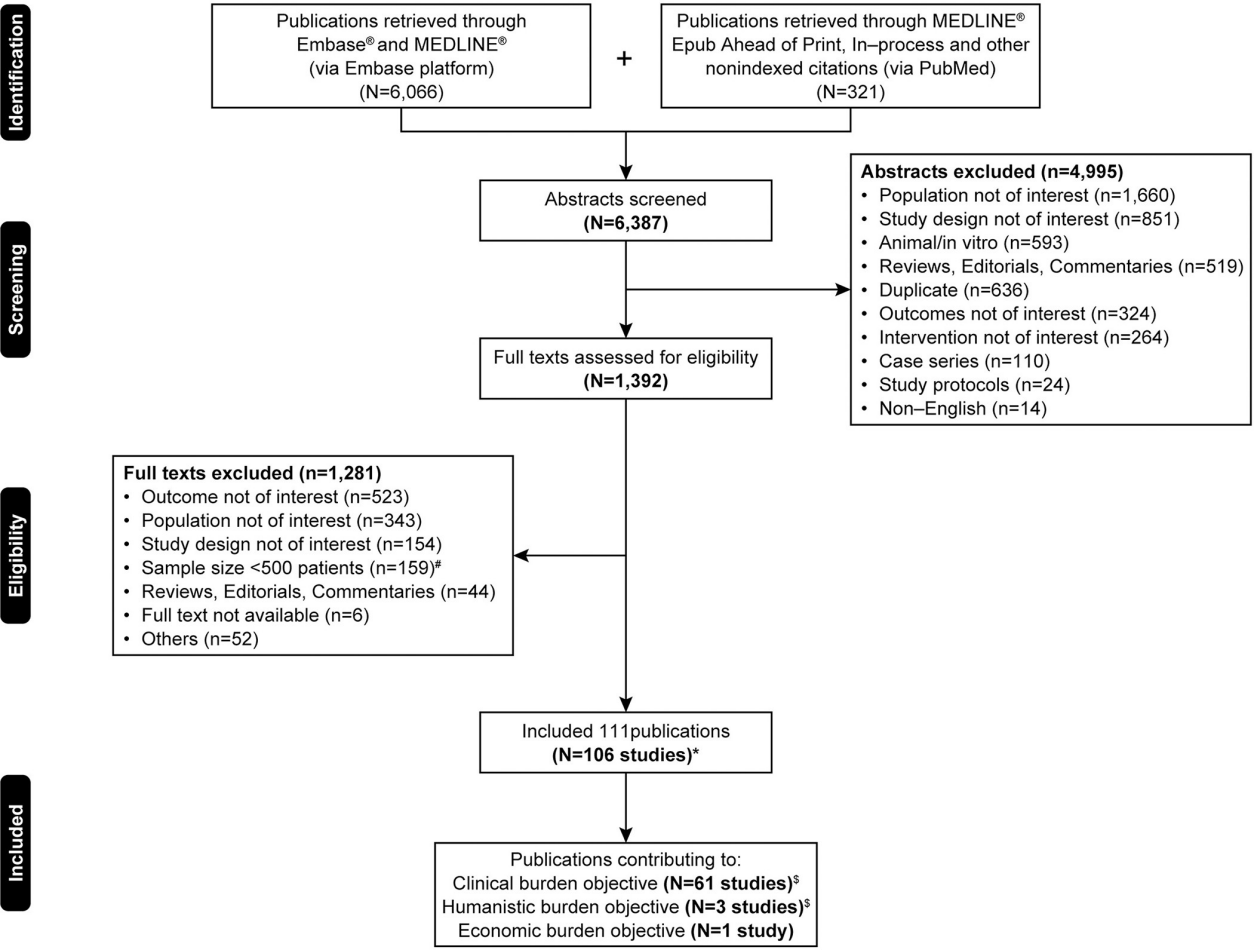
PRISMA flow diagram. PRISMA: Preferred Reporting Items for Systematic Reviews and Meta-Analyses. *Includes three linked studies. ^$^These objectives are reported in overlapping studies. ^#^Exclusion criteria of sample size <500 was applied for the clinical burden objective only.

This SLR included 106 studies (from 111 publications, including three linked studies), of which 61 (including two linked studies) reported the clinical burden of elevated Lp(a) in patients with ASCVD (Fig 1). Twenty-six studies reported clinical burden of clinically relevant elevated Lp(a) in patients with ASCVD. Of the 26 studies, 46% were from Asia (n = 12), 35% were from Europe (n = 9), 8% were from the North America (n = 2), 4% were from Oceania (n = 1), and 8% were multinational (n = 2, including one multinational study from Europe). The study design of the included studies was prospective observational (n = 20; 77%), retrospective observational (n = 5; 19%), and RCTs (n = 1; 4%). The follow-up duration of the studies varied from 1 year to 11 years. The sample size of the studies ranged from 536 to 413,734. One of the 26 studies with a retrospective study design from China included for clinical burden also provided data for economic burden (sample size: 944 and median follow-up: 1.4 years). Additionally, two prospective observational studies with 1 year follow-up duration and a single cross-sectional study from China reported data for humanistic burden (sample size range: 456 to 9709). The characteristics of the included studies are summarized in Table 1.

**Table 1:** Study characteristics of studies included for clinical, humanistic, and economic burden among patients with ASCVD and elevated Lp(a).

Study name	Region	Country	Study design	Median follow-up duration (years)	Population	N (study sample size)	n (sample size for elevated Lp(a))	Lp(a) threshold (mg/dL)
**Clinical burden**								
Galasso 2021 [19]	Europe	Italy	RCS	2.2	ACS	724	242	≥30
Gao 2021 [20]	Asia	China	PCS	3.5	ACS	1,179	350	≥30
Liu 2021 [21]	Asia	China	PCS	4.57*	ASCVD	8,668	2,588	≥30
Sang 2021 [22]	Asia	China	RCS	5.5	ACS	536	NR	≥30
Wu 2021 [23]	Asia	China	RCS	NR	ACS	1,292	726	≥30
Golledge 2020 [24]	Oceania	Australia	PCS	2.4	PAD	1,472	529	≥30
Liu 2020 [25]	Asia	China	PCS	4.9*	ASCVD	4,078	1,247	≥30
Zhang M 2020 [26]	Asia	China	PCS	3^#^	ACS	1,008	336	≥30
Zhang 2020 [27]	Asia	China	PCS	3	ASCVD	8,417	NR	≥30
Zhu 2021 [28]	Asia	China	PCS	2.1	ACS	6,601	2,285	≥30
Cai 2019 [29]	Asia	China	PCS	NR	ASCVD	2,517	432	≥30
Gencer 2019 [30]	Europe	Switzerland	PCS	1^^^	ACS	1,711	276	≥30
Nicholls 2010 [31]	North America	USA	PCS	3^^^	ASCVD	2,769	1,049	≥30
Yoon 2021 [32]	Asia	Korea	PCS	7.4	Revascularization	12,064	3,747/1,777	≥30/≥50
Bigazzi 2021 [33]	Europe	Italy	PCS	2*	ASCVD	2,374	443	≥50
Sanchez Muñoz-Torrero 2018 [34]	Europe	Spain	PCS	3*	ASCVD	1,503	370	≥50
Puri 2017^~^ [35]	Multiple	Multiple	RCT	2^^^	ASCVD	915	239	≥50
Wong 2021 [36]	North America	USA and Canada	PCS	3.3*	ASCVD	3,359	536	≥70
Wang 2020 [37]	Asia	China	RCS	2	ACS	1,464	NR	>75^@^
Arnold 2021^∫^ [38]	Europe	Multiple	PCS	1	Stroke	1,733	285	≥100^@^
Wohlfahrt 2021 [39]	Europe	Czech Republic	PCS	1.6	ACS	851	149	≥125^@^
Patel 2020 [14]	Europe	UK	PCS	11.2	ASCVD	17,326	3,510	≥150^@^
Welsh 2020 [40]	Europe	UK	PCS	9	ASCVD	413,734	18,731/15,996/13,095	≥100/≥125/≥150^@^
Madsen 2020 [41]	Europe	Denmark	PCS	5	ASCVD	2,527	212	≥200^@^
Waissi 2020 [42]	Europe	The Netherlands	PCS	2.6*	ASCVD	944	189	≥200^@^
**Clinical and economic burden**								
Yang 2022 [43]	Asia	China	RCS	1.4	ACS	944	203	≥30
**Humanistic burden**								
Bao 2021 [44]	Asia	China	CSS	NR	ASCVD	456^±^	NR^¥^	NR^€^
Jiang 2021 [45]	Asia	China	PCS	1^^^	Stroke	9,709	2,427	>35.8
Li 2021^$^ [46]	Asia	China	PCS	1^^^	Stroke	1,017	255	Quartile 4^$^

ACS: Acute coronary syndrome; ASCVD: Atherosclerotic cardiovascular disease; CSS: Cross-sectional study; Lp(a): Lipoprotein(a); NR: Not reported; PAD: Peripheral artery disease; PCS: Prospective cohort study; RCS: Retrospective cohort study; RCT: Randomized controlled trial; UK: United Kingdom; USA: United States of America.

*Represents mean values

^#^represents average values, and

^^^represents mean or median not mentioned/reported; ^@^ represents nmol/L.

^$^In Li 2021 [46], the Lp(a) threshold for quartile 4 (Q4) is not mentioned.

^~^Puri 2017 [35] involves multiple countries, including the USA, Canada, and Australia.

^∫^Arnold 2021 [38] involves multiple countries, including Switzerland, Greece, Spain, and Germany.

^±^Bao 2021 [44] was included for providing evidence for humanistic burden despite having a sample size of <500, due to paucity of evidence.

^¥^Lp(a) was measured in 226 male premature chronic heart disease (PCHD) patients. The sample size of patients with elevated Lp(a) was not reported.

^€^Lp(a) was considered as a continuous variable and the thresholds for elevated Lp(a) were not reported.

### Patient characteristics

Thirteen of the twenty-six studies reported clinical burden for ASCVD, ten studies reported data for ACS, and one study each reported data for peripheral artery disease (PAD), stroke, and revascularization. Fifteen studies defined elevated Lp(a) levels as ≥30 mg/dL, four as ≥50 mg/dL, and one as ≥70 mg/dL. No studies were available for elevated Lp(a) defined as ≥90 or ≥180 mg/dL or super-elevated Lp(a) defined as ≥300 mg/dL. One study defined elevated Lp(a) as >75 nmol/L, while two studies defined elevated Lp(a) level as ≥100/≥125/≥150 nmol/L and super-elevated Lp(a) level as ≥200 nmol/L. No studies were available for elevated Lp(a) defined as ≥90 nmol/L or super-elevated Lp(a) defined as ≥400/≥430 nmol/L. Seventeen of the twenty-six studies (including one study that reported data for economic burden) reported patient characteristics for patients with elevated Lp(a) levels. In these studies, the mean age ranged from 56.6 to 71.3 years. All studies reported a higher proportion of male patients (53%–79%) and patients with hypertension (54%–89%). All studies reported a lower proportion of patients with diabetes mellitus (3.5%–35%). Only four studies reported varying proportion of patients with dyslipidemia (12%–58.8%). Five studies reported the proportion of patients with a family history of CVD (15%–68%). High variability was observed in the measurement units for CV biochemical markers, including different lipids, hemoglobin A1c (HbA1c), and high-sensitivity C-reactive protein (hsCRP). Nine of the twenty-six studies included for clinical burden did not report patient characteristics [19,22,27,35–38,40,42].

In a single study that reported the economic burden and clinical burden, the mean age of ACS patients with elevated Lp(a) levels (≥30 mg/dL) was 65.5 years. The study had a higher proportion of male patients (77%) and patients with hypertension (62%) [43].

Two of the three studies contributing to humanistic burden reported patient characteristics; however, one study, where Lp(a) was reported as a continuous variable, did not report patient characteristics [44]. In the former studies, stroke patients with elevated Lp(a) levels (defined as ≥35.8 mg/dL by Li et al. [46] and as quartile 4 by Jiang et al. [45]) had a higher proportion of male patients (68%–77%) and patients with hypertension (63%–78%) [45,46]. The median age of the patients ranged from 61 to 63 years. The characteristics of the included studies are summarized in Table 2.

**Table 2:** Patient characteristics of studies included for clinical, humanistic, and economic burden among patients with ASCVD and elevated Lp(a).

Study name	Country	Population	Lp(a) threshold (mg/dL)	N	Age (y) (mean)	Male (%)	Comorbidities (%)			Clinical laboratory values (mean)						Family history (%)
							DLP	HTN	DM	HDL-C (mg/dL)	LDL-C (mg/dL)	TC (mmol/L)	TG (mmol/L)	HbA1c	hsCRP (mg/L)	
**Clinical burden**																
Gao 2021 [20]	China	ACS	≥30	350	56.6	71.1	58.8	60.6	21.8	42.9	91.6	155^^^	126.6*^^^	5.9	23.3*^^^	NR
Liu 2021 [21]	China	ASCVD	≥30	2588	57.6	68.9	NR	59.8	26.1	1.07^#^	2.71^#^	4.33	1.43*	6.3	1.47*	NR
Wu 2021 [23]	China	ACS	≥30	726	57.1	74.4	54.8	65.4	34.6	1.1^#^	3.1^#^	5.0	1.9*	5.9	NR	NR
Golledge 2020 [24]	Australia	PAD	≥30	529	71.3	78.8	NR	78.6	28	1.2*^@^	2.4*^#^	4.4*^@^	1.4*^@^	NR	NR	NR
Liu 2020 [25]	China	ASCVD	≥30	1247	57.0	72.3	NR	60.9	26	1.07^#^	2.66	4.28	1.47*	6.28	1.47	15.2
Zhang M 2020 [26]	China	ACS	≥30	336	82.9	65.8	NR	88.7	3.5	1.1^#^	2.5^#^	4.08	1.27*	NR	7.61	NR
Zhu 2021 [28]	China	ACS	≥30	2285	59.0	74.4	NR	64.9	29	1.05^#^	2.66	4.36	1.67	NR	3.4	25.3
Cai 2019 [29]	China	ASCVD	≥30	432	61.6	58.6	NR	60.9	15.3	1.19^#^	2.59^#^	4.48	1.29	NR	NR	NR
Gencer 2019 [30]	Switzerland	ACS	≥30	276	64.1	68.8	NR	57.6	16	1.3^#^	3.7^#^	5.1	1.1	NR	9.5	29.2
Nicholls 2010 [31]	USA	ASCVD	≥30	1049	63.0	68.6	NR	85.9	25.8	34.8	10.0	NR	119*^^^	NR	2.4*^^^	NR
Yoon 2021 [32]	Korea	Revascularization	≥30	3747	62.4	68.9	49.6	59.1	31.5	NR	NR	NR	NR	NR	NR	NR
Bigazzi 2021 [33]	Italy	ASCVD	≥50	443	66.3	66.0	NR	53.7	18.3	44.6	125.4	193.3^^^	120.8^^^	NR	NR	NR
Sanchez Muñoz-Torrero 2018 [34]	Spain	ASCVD	≥50	370	66.0	74.0	NR	62.0	35	48	105.0	180.0^^^	129.0^^^	6.0*	NR	NR
Wohlfahrt 2021 [39]	Czech Republic	ACS	≥125^@^	149	65.0	73.8	NR	61.7	21.5	1.18^#^	3.19^#^	4.76	1.3*	NR	NR	28.9
Patel 2020 [14]	UK	ASCVD	≥150^@^	3510	62.4	75.2	NR	71.8	16.2	NR	109.5	NR	NR	NR	NR	67.7
Madsen 2020 [41]	Denmark	ASCVD	≥200^@^	212	68*	53	NR	83	17	NR	100*	NR	145*^^^	NR	NR	NR
**Clinical and economic burden**																
Yang 2022 [43]	China	ACS	≥30	203	65.5	76.6	12.0	62.1	22.7	1.14*^#^	2.52*^#^	4.23*	1.15*	NR	NR	NR
**Humanistic burden**																
Jiang 2021 [45]	China	Stroke	> 35.8	2427	63*	67.7	8.8	62.8	23.5	1.1*^&^	2.6*^&^	NR	1.3*^&^	NR	2*	NR
Li 2021^$^ [46]	China	Stroke	Quartile 4^$^	255	61*	76.5	12.6	78.4	37.3	0.93*^#^	2.43*^#^	3.97*	1.2*	NR	NR	NR

ACS: Acute coronary syndrome; ASCVD: Atherosclerotic cardiovascular disease; DLP: Dyslipidemia; DM: Diabetes mellitus; HbA1c: Hemoglobin A1c; HDL-C: High-density lipoprotein cholesterol; hsCRP: High-sensitivity C-reactive protein; HTN: Hypertension; LDL-C: Low-density lipoprotein cholesterol; Lp(a): Lipoprotein(a); NR: Not reported; TC: Total cholesterol; TG: Triglycerides; UK: United Kingdom; USA: United States of America.

Familial hypercholesterolemia (19.9%) is reported in one study (Gencer 2019) [30] and hypercholesterolemia (81%) is reported in one study (Waissi 2020) [42].

^$^Lp(a) threshold for Q4 is not mentioned.

Patient characteristics were not available for Arnold 2021 [38], Galasso 2021 [19], Wong 2021 [36], Waissi 2020 [42], Wang 2020 [37], Welsh 2020 [40], Bao 2021 [44], Zhang 2020 [26], and Puri 2017 [35]. Patient characteristics are also not available for patients with Lp(a) levels ≥50 mg/dL for Yoon 2021 [32].

*Represents median values

^#^represents units in mmol/L

^^^represents units in mg/dL

^@^represents units in nmol/L; and

^&^represents units in mM.

### Clinical burden

#### MACE

**Fig 2:**
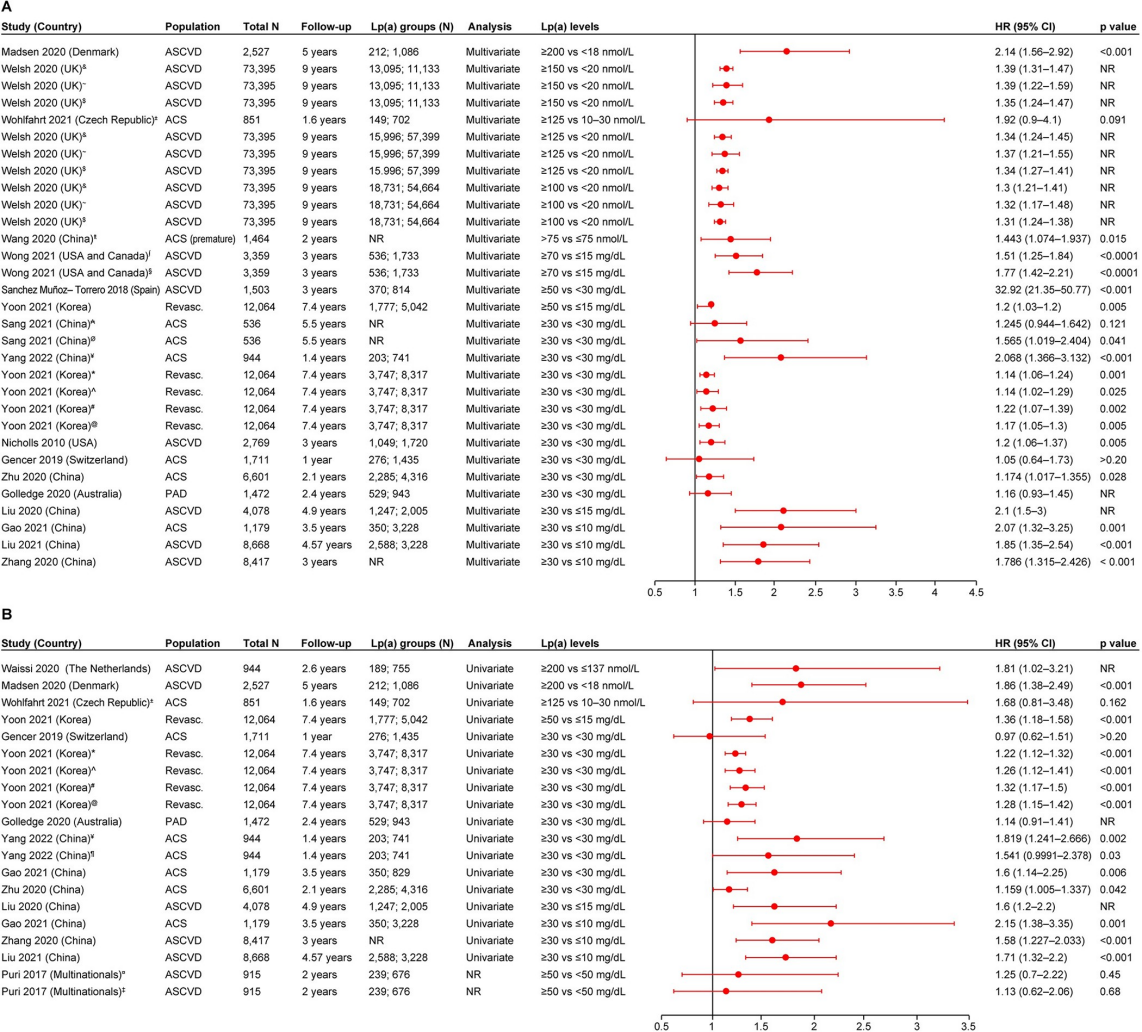
HR (95% CI) for the association of Lp(a) and MACE. A. Results of multivariate analysis and B. Results of univariate analysis or from studies where analysis type was not reported. ACS: Acute coronary syndrome; ASCVD: Atherosclerotic cardiovascular disease; CI: Confidence interval; CV: Cardiovascular; CVD: Cardiovascular disease; HR: Hazard ratio; Lp(a): Lipoprotein(a); MACE: Major adverse cardiac event; MI: Myocardial infarction; NR: Not reported; PAD: Peripheral artery disease; Revasc.: Revascularization; UK: United Kingdom; USA: United States of America. Yoon 2021 [32] reported four definitions for MACE and for each definition, results of univariate and multivariate analysis are reported: *CV death with spontaneous MI, ischemic stroke, and any repeat revascularization; ^^^The composite of CV death and spontaneous MI; ^#^The composite of CV death, excluding unknown cause of death, spontaneous MI, and ischemic stroke; and ^@^Primary outcomes: the composite of CV death, spontaneous MI, and ischemic stroke. Welsh 2020 [40] reported three definitions for MACE outcomes: ^&^Coronary heart disease, ^~^Fatal CVD, and ^$^Composite CVD outcome. ^₶^Wang 2020 [37] defined MACE as cardiac death and ACS, including unstable angina pectoris and acute MI (AMI). ^±^Wohlfahrt 2021 [39] defined MACE as recurrent cardiovascular events (CVE), including hospitalization for ACS, ACS recurrence, or death from CV causes. Wong 2021 [36] reported two definitions for MACE outcomes: ^∫^Total ASCVD event and ^§^First ASCVD event. ^¥^Analysis was done using Cox proportional hazards model. ^¶^Analysis was done using log-rank test. ^Ø^Represents hard chronic heart disease (CHD) events. Sang 2021 [22] reported two definitions for MACE outcomes: ^₳^represents the MACE outcomes and ^Ø^represents hard CHD events. Puri 2017 [35] reported two definitions for MACE outcomes: ^¤^defined as changes in MACE, death, non-fatal MI, stroke, coronary revascularization, or hospitalization for unstable angina and ^‡^defined as death, non-fatal MI, stroke, coronary revascularization, or hospitalization for unstable angina.

MACE as the clinical outcome in patients with ASCVD was reported in 20 studies. The definition of MACE varied across the included studies. Ten studies were from Asia, six were from Europe, two were from North America, one was from Oceania, and one was a multinational study. Nineteen studies reported HRs, three studies reported the events percentage of MACE, two studies reported the event rates for MACE, one study reported the OR, and one study reported the RR. The risk of MACE associated with elevated vs lower Lp(a) levels was shown to be significantly greater in 14 of the 16 studies reporting HRs from multivariate analyses. The HRs in these studies ranged from 1.14 (95% CI, 1.02–1.29) to 32.92 (95% CI, 21.35–50.77) [20–22,25,27,28,31,32,34,36,37,40,41,43]. Three of the 16 studies reporting HRs from multivariate analyses showed a trend for association between elevated Lp(a) levels and MACE vs low Lp(a) levels; however, the association was not significant (Fig 2A) [22,24,30]. Twelve studies reported HRs from univariate analyses (Fig 2B). Eight of 12 studies reported a positive association between elevated Lp(a) levels and MACE vs low Lp(a) levels (HR [95% CI] range: 1.22 [1.12–1.32] to 1.86 [1.38–2.49]) [20,21,25,27,28,32,41,42]. Of the remaining four studies, one study showed positive association for MACE in the Cox proportional model, while it showed a trend for association in the log-rank test [43]. Two studies showed a trend for association between elevated Lp(a) levels and MACE (however, the results were not significant) [24,39], while the other study showed no association between the variable and the outcome [30]. The single RCT also reported a trend for association between elevated Lp(a) levels (≥50 mg/dL) and MACE vs low Lp(a) levels (<50 mg/dL); however, the association was not significant. For this study, the analysis type (i.e., univariate or multivariate) was not reported [35] (Fig 2B).

Three studies reported significantly higher events percentage of MACE (ranging from 5.6%–41.8%) in patients with ASCVD and ACS with elevated Lp(a) levels (≥30 mg/dL) vs low Lp(a) levels (<30 or <10 mg/dL; ranging from 3.5%–35.8%) [27,31,43]. One of the two studies reporting the event rates for MACE reported a numerically higher rate of MACE in patients with symptomatic artery disease and elevated Lp(a) (≥50 mg/dL; 14 per 100 patient-years) vs patients with low Lp(a) levels (<30 mg/dL; 2.87 per 100 patient-years; p value was not reported) [34]. The remaining one study reported similar event rates of MACE for patients with elevated and low Lp(a) levels [32]. Wu et al. reported a 28% increased odds of MACE (i.e., acute stent thrombosis, MI, ischemic stroke or transient ischemic attack (TIA), congestive heart failure, and CV mortality) in patients with ACS with elevated Lp(a) levels (≥30 mg/dL) vs patients with low Lp(a) levels (<30 mg/dL) (OR [95% CI]: 1.28 [1.18–2.42]) [23]. In the study by Sanchez Muñoz-Torrero et al., patients with symptomatic artery disease and an Lp(a) level of ≥50 mg/dL had a two-fold increased risk of having MACE (i.e., subsequent ischemic events and death) over a 3-year period vs those with Lp(a) <30 mg/dL (RR [95% CI]: 2.06 [1.73–2.45]) [34].

#### CV mortality and all-cause mortality

**Fig 3:**
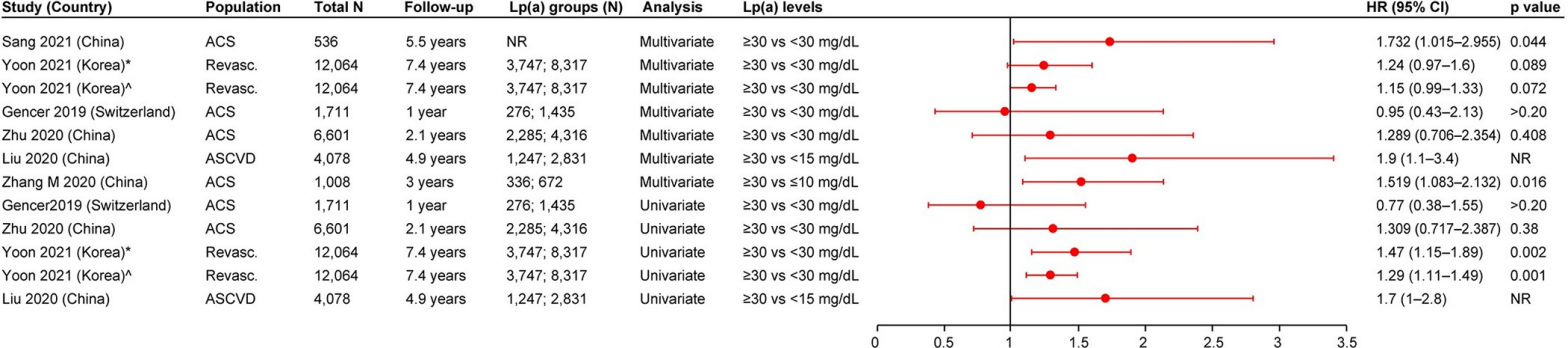
HR (95% CI) for the association of Lp(a) and CV mortality. ACS: Acute coronary syndrome; ASCVD: Atherosclerotic cardiovascular disease; CI: Confidence interval; CV: Cardiovascular; HR: Hazard ratio; Lp(a): Lipoprotein(a); NR: Not reported. Yoon 2021 [32] provided two definitions for CV death: *CV death, excluding unknown cause of death and ^^^CV death was any death from a CV cause.

Nine studies provided data for the outcome of CV mortality in patients with ASCVD. Six studies were from Asia, two were from Europe, and one was from Oceania. While eight studies reported the events percentage of CV mortality, six studies reported the HRs, and one study reported the OR. All six studies reporting HR defined elevated Lp(a) as ≥30 mg/dL. In three of the six studies providing HRs from multivariate analyses, elevated Lp(a) levels were significantly associated with an increased risk of CV mortality, with HRs ranging from 1.519 (95% CI, 1.083–2.132) to 1.9 (95% CI, 1.1–3.4) vs low Lp(a) levels [22,25,26]. Of the three remaining studies, two showed a trend for positive association between elevated Lp(a) levels and CV mortality (however, the results were not significant) [28,32], while one study showed no association [30]. Four studies reported the HRs from univariate analyses. One of these four studies reported a significant positive association between CV mortality and elevated Lp(a) levels vs low Lp(a) levels (HR [95% CI] range: 1.29 [1.11–1.49] to 1.47 [1.15–1.89]) [32], while two studies showed a trend for positive association (however, the results were not significant) [25,28]. The remaining one study reported no association between elevated Lp(a) and CV mortality [30] (Fig 3). The single study reporting OR demonstrated no association between elevated Lp(a) (≥30 mg/dL) and CV mortality in patients with ACS vs patients with low Lp(a) levels (<30 mg/dL) [23]. Eight studies reported the events percentage of CV mortality. One of the eight studies reported significantly higher CV mortality in patients with acute MI with elevated Lp(a) levels (>30 mg/dL) vs patients with low Lp(a) levels (≤10 mg/dL) (31.8% vs 22.8%) [26]. In seven of the eight studies, no trend or association was reported [23–25,28,30,32,34].

**Fig 4:**
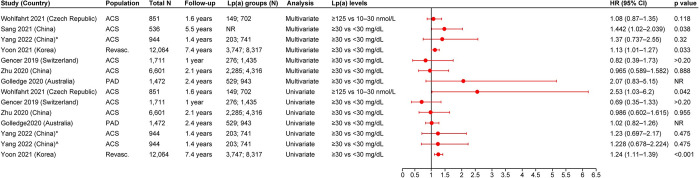
HR (95% CI) for the association of Lp(a) and all-cause mortality. ACS: Acute coronary syndrome; CI: Confidence interval; HR: Hazard ratio; Lp(a): Lipoprotein(a); NR: Not reported; PAD: Peripheral artery disease; Revasc.: Revascularization. *Analysis was done using Cox proportional hazards model. ^^^Analysis was done using log-rank test.

All-cause mortality in patients with ASCVD and elevated Lp(a) were reported in ten studies (including five studies from Asia, three from Europe, one from North America, and one from Oceania). Of the ten studies, seven reported HRs and seven reported the events percentage. Five of the seven studies reporting HRs from multivariate analyses showed a trend for positive association between elevated Lp(a) levels and all-cause mortality (however, the association was not significant) [22,24,32,39,43], while two studies did not show an association [28,30]. One of the six studies reporting HRs from univariate analyses showed a significant association between elevated Lp(a) levels (>125 nmol/L vs 10–30 nmol/L) and all-cause mortality (HR [95% CI]: 2.53 [1.03–6.2]) [39]. Three of the six studies reporting HRs from univariate analyses showed a trend for positive association between elevated Lp(a) levels and all-cause mortality (however, the association was not significant) [24,32,43], while two studies did not show an association [28,30] (Fig 4).

One of the seven studies reporting all-cause mortality events percentage reported significantly higher events percentage in patients with MI with nonobstructive coronary arteries (MINOCA) and elevated Lp(a) levels (≥30 mg/dL) vs patients with MINOCA and low Lp(a) levels (<10 mg/dL) during the 3-year follow-up (3.1% vs 0.5%) [20]. Additionally, three of the seven studies reported numerically higher all-cause mortality events percentage in patients with elevated Lp(a) levels; however, the results did not reach statistical significance [31,34,43]. The three remaining studies reported inconclusive results [24,28,30].

#### MI

**Fig 5:**
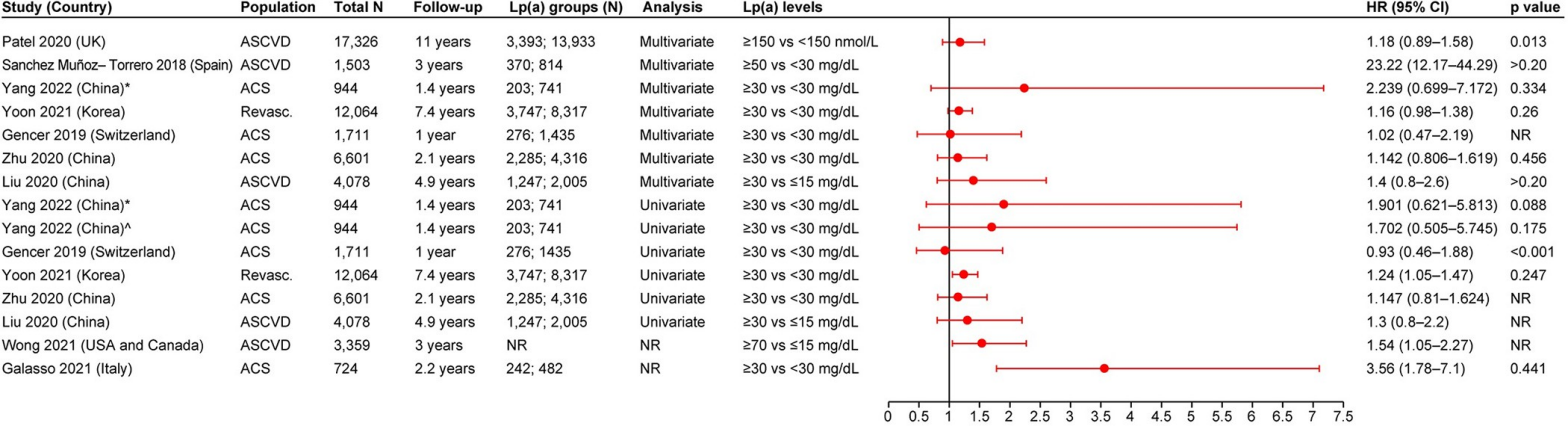
HR (95% CI) for the association of Lp(a) and MI. ACS: Acute coronary syndrome; ASCVD: Atherosclerotic cardiovascular disease; CI: Confidence interval; HR: Hazard ratio; Lp(a): Lipoprotein(a); MI: Myocardial infarction; NR: Not reported; Revasc.: Revascularization; UK: United Kingdom; USA: United States of America. *Analysis was done using Cox proportional hazards model. ^^^Analysis was done using log-rank test.

Eleven studies reported MI as an outcome in patients with ASCVD. Six studies were from Asia, four were from Europe, and one was from North America. Nine studies reported the HRs, two reported the rates of MI, and two reported the ORs. Compared with patients with lower Lp(a) levels, patients with elevated Lp(a) levels had a greater risk of MI in the seven studies reporting HRs from multivariate analyses. Of these seven studies, one study reported a significant association (HR [95% CI]: 23.22 [12.17–44.29]) [34], while the remaining six showed a trend for association between elevated Lp(a) levels and MI, although the results did not reach statistical significance [14,25,28,30,32,43]. Five studies reported HRs from univariate analyses. One of the five studies showed a significant association between elevated Lp(a) levels (≥30 mg/dL) and MI vs low Lp(a) levels (<30 mg/dL) [32], while, three studies showed a trend for positive association (however, the results were not significant) [25,28,43]. The remaining one study showed no association between Lp(a) levels and MI [30]. Additionally, two studies for which the analysis type was not reported showed a significant association between elevated Lp(a) levels and an increased risk of MI (HR [95% CI] range: 1.54 [1.05–2.27] to 3.56 [1.78–7.1]) [19,36] (Fig 5). Two studies reported the OR for the association between elevated Lp(a) levels and MI. One of these studies reported a 52.1% increased odds of MI with elevated Lp(a) levels (≥30 mg/dL) vs low Lp(a) levels (<30 mg/dL) (OR [95% CI]: 1.521 [1.179–1.963] [29], while one study showed a trend for association (however, the association was not significant) [23]. One of the two studies reporting the event rates for MI reported a numerically higher rate of MI in patients with symptomatic artery disease and elevated Lp(a) (≥50 mg/dL; 4.68 per 100 patient-years) vs patients with low Lp(a) levels (<30 mg/dL; 1.36 per 100 patient-years; p value was not reported) [34]. The remaining one study reported similar event rates of MI for patients with elevated and low Lp(a) levels [32].

#### Stroke

**Fig 6:**
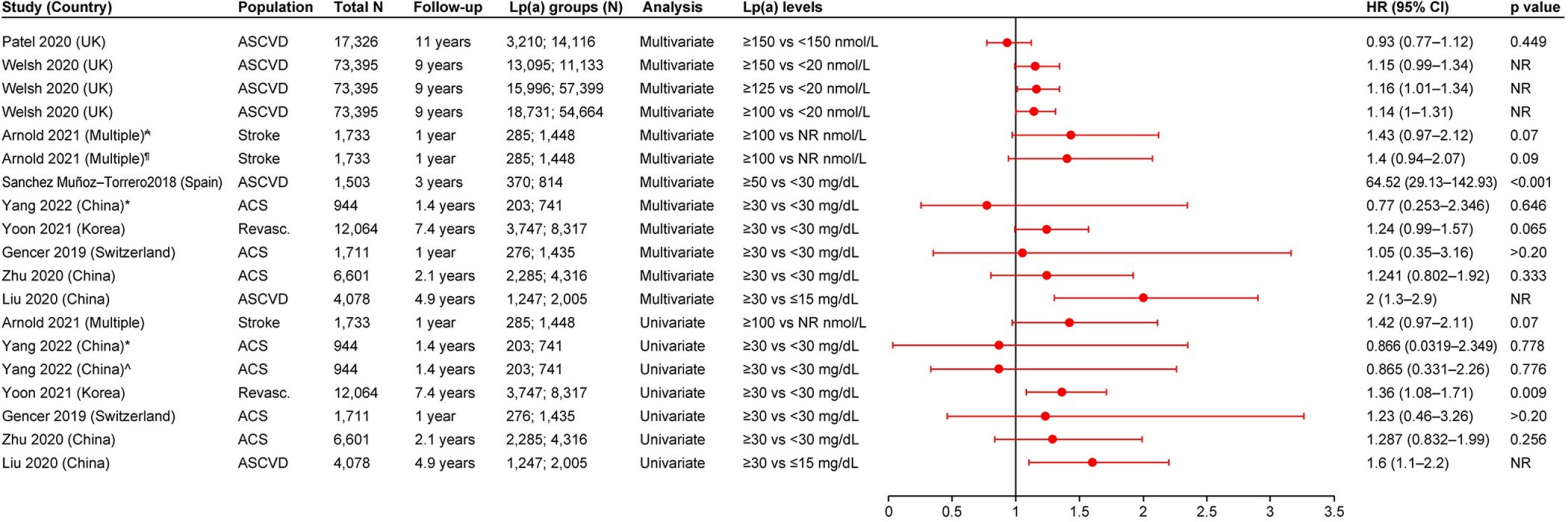
HR (95% CI) for the association of Lp(a) and stroke. ACS: Acute coronary syndrome; ASCVD: Atherosclerotic cardiovascular disease; CI: Confidence interval; HR: Hazard ratio; Lp(a): Lipoprotein(a); NR: Not reported; Revasc.: Revascularization; UK: United Kingdom. *Analysis was done using Cox proportional hazards model. ^^^Analysis was done using log-rank test. ^₳^Adjusted for age and sex. ^¶^Adjusted for vascular risk factors, including dyslipidemia, hypertension, smoking status, diabetes, and previous stroke.

The association of Lp(a) with stroke in patients with ASCVD was reported in ten studies. Five studies were from Asia and five were from Europe (including one multinational study from Europe). Of these ten studies, nine reported HRs, two reported rates of stroke, and one reported OR. Three of the nine studies reporting HRs from multivariate analyses showed significant association of elevated Lp(a) levels with stroke vs lower Lp(a) levels (HR [95% CI] range: 1.16 (1.01–1.34] to 64.52 [29.13–142.93]) [25,34,40]. In most of the remaining studies, a trend for increasing risk of stroke was observed with elevated Lp(a) levels; however, the association was not significant [28,30,32,38,40]. Two studies did not report significant results or show an increasing trend for association [14,43]. Six studies reported the HRs from univariate analyses. Two of the six studies reported a significant association between elevated Lp(a) levels and an increased risk of stroke (HR [95% CI] range: 1.36 [1.08–1.71] to 1.6 [1.1–2.2]) [25,32]. Three of the remaining studies showed a trend for association between elevated Lp(a) levels and stroke (however, the results did not reach statistical significance) [28,30,38], while one showed no association [43] (Fig 6). The single study reporting OR showed increased odds of stroke in patients with elevated Lp(a) levels (≥30 mg/dL) vs patients with low Lp(a) levels (<30 mg/dL); however, the association was not significant [23]. One of the two studies reporting the event rates for stroke reported a numerically higher rate of stroke in patients with symptomatic artery disease and elevated Lp(a) levels (≥50 mg/dL; 6.88 per 100 patient-years) vs patients with low Lp(a) levels (<30 mg/dL; 0.89 per 100 patient-years; p value was not reported) [34]. The remaining one study reported similar event rates of stroke for patients with elevated and low Lp(a) levels [32].

#### Revascularization

**Fig 7:**
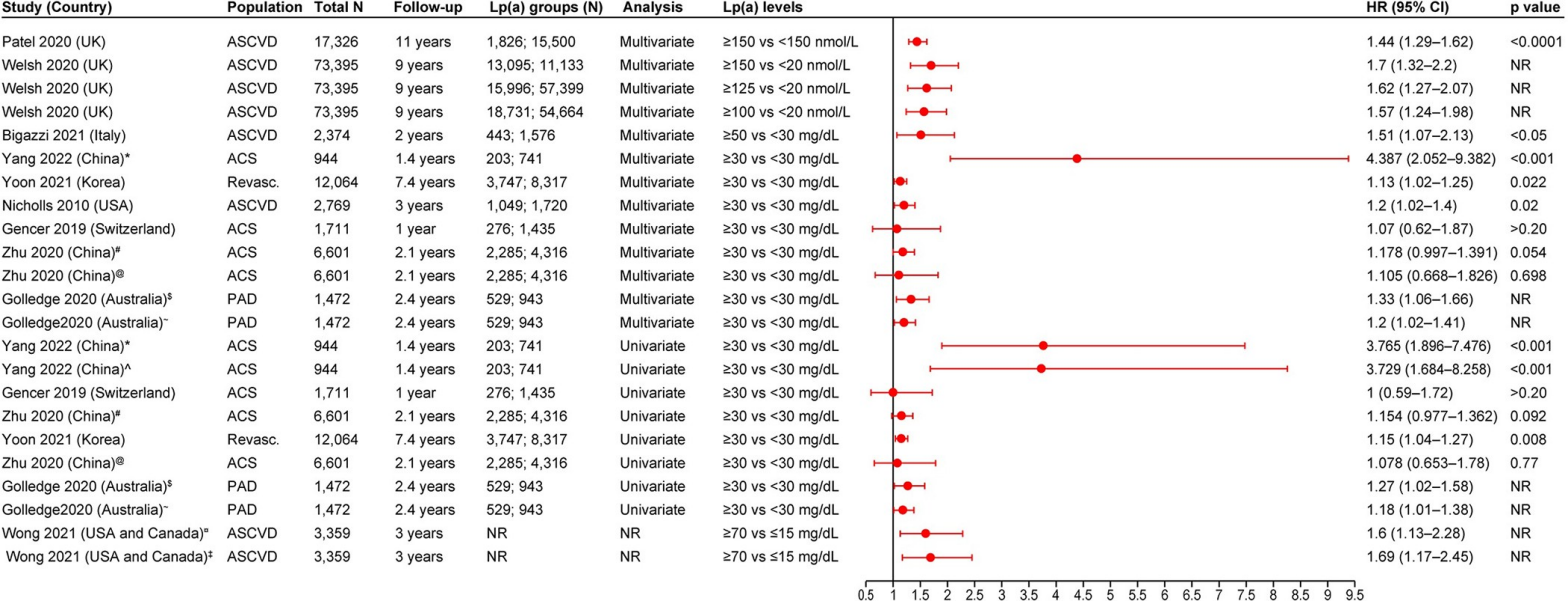
HR (95% CI) for the association of Lp(a) and revascularization. ACS: Acute coronary syndrome; ASCVD: Atherosclerotic cardiovascular disease; CI: Confidence interval; HR: Hazard ratio; Lp(a): Lipoprotein(a); NR: Not reported; PAD: Peripheral artery disease; PCI: Percutaneous coronary intervention; Revasc.: Revascularization; UK: United Kingdom; USA: United States of America. Zhu 2020 [28] reported two definitions for revascularization: ^#^Unplanned target vessel revascularization was defined as any repeat PCI or coronary artery bypass grafting (CABG) and ^@^Patients with stent stenosis. Golledge 2020 [24] reported two definitions for revascularization: ^$^Patients undergoing lower limb peripheral revascularization (open or endovascular) and ^~^Any PAD operation, including the lower limb (open and endovascular) peripheral revascularization, carotid artery revascularization, open and endovascular abdominal aortic aneurysm repair, and other aneurysm repair. *Analysis was done using Cox proportional hazards model. ^^^Analysis was done using log-rank test. Wong 2021 [36] reported two definitions for revascularization: ^¤^Cerebral revascularization and ^‡^ Total coronary revascularization.

Ten studies reported revascularization outcome in patients with ASCVD (including four studies from Europe, three from Asia, two from North America, and one from Australia). While all studies reported the HRs, two reported events percentage and one reported the rates of revascularization. Seven of the nine studies reporting HRs from multivariate analyses showed significantly higher risk of revascularization among patients with elevated Lp(a) levels, with HRs ranging between 1.13 (95% CI, 1.02–1.25) and 4.387 (95% CI, 2.052–9.382) [14,24,31–33,40,43]. The two remaining studies showed a trend for association; however, the association was not significant [28,30]. Three of the five studies reporting HRs from univariate analyses showed a significant positive association between elevated Lp(a) levels (≥30 mg/dL) and an increased risk of revascularization (HR [95% CI] range: 1.15 [1.04–1.27] to 3.765 [1.896–7.476]) vs low Lp(a) levels (<30 mg/dL) [24,32,43]. Two of the five studies showed a trend for association between elevated Lp(a) levels and revascularization; however, the association was not significant [28,30]. Additionally, one study with unknown analysis type reported a significant positive association between elevated Lp(a) levels (≥70 mg/dL) and an increased risk of revascularization (HR [95% CI] range: 1.6 [1.13–2.28] to 1.69 [1.17–2.45]) vs low Lp(a) levels (≤15 mg/dL) [36] (Fig 7). Two studies reporting results for revascularization events percentage showed numerically higher events percentage for patients with elevated Lp(a) levels (8.9–30.9%) vs patients with low Lp(a) levels (3%–26%; p values were not reported) [31,43]. A single study reported similar event rates (per 100 person-years) of revascularization for patients with elevated and low Lp(a) levels [32].

### Humanistic burden

Three studies reported data for humanistic burden. In a prospective cohort study from China, patients with acute ischemic stroke (AIS) or TIA from the Third China National Stroke Registry (CNSR-III) with the highest Lp(a) quartile had higher events percentage of cognitive impairment (defined as MoCA ≤22) at 1 year vs those with the lowest quartile of Lp(a) (35.7% vs 25.5%; p value was not reported). Patients in the highest Lp(a) quartile were at significantly higher odds of cognitive impairment vs patients in the lowest Lp(a) quartile (unadjusted OR [95% CI]: 1.62 [1.11–2.37]. Results from multivariate analyses showed similar trend; however, the results were not statistically significant. In contrast, no association was found between Lp(a) levels and cognitive improvement (MoCA increase by ≥20% or ≥30%) from 2 weeks to 1 year from discharge after AIS or TIA [46]. Another study on patients with AIS or TIA from the CNSR-III reported that elevated Lp(a) levels were significantly associated with increased disability related to stroke (evaluated by an mRS score ≥3) at 3 months and 1 year (OR [95% CI] for quartile 4 [>35.8 mg/dL] vs quartile 1 [<8.9 mg/dL]:1.58 [1.34–1.86] and 1.46 [1.23–1.72], respectively) [45]. In a cross-sectional study from China that included 226 male patients with PCHD, Spearman’s multiple linear regression analysis showed that the MCS score negatively correlated with Lp(a) levels after adjusting for risk factors (R = −0.295, p<0.001). The PCS score did not correlate with Lp(a) levels (R = 0.069, p = 0.314). Lp(a) levels weakly correlated with HRQoL. With an increase in the Lp(a) levels, the HRQoL, as assessed by SF-36, decreased (R = −0.166, p = 0.014) [44].

### Economic burden

A retrospective observational study from China that included patients with ACS reported no difference in median length of hospital stay in days for patients with elevated Lp(a) levels (≥30 mg/dL) and those with low Lp(a) levels (<30 mg/dL). The study also reported slightly higher median hospitalization costs (in 10,000 yuan) for patients with elevated Lp(a) levels (≥30 mg/dL) vs patients with low Lp(a) levels (<30 mg/dL) (3.83 vs 3.52; the result was not significant) [43].

### Feasibility assessment for meta-analysis

The meta-analysis feasibility assessment was done for 61 studies that provided data for clinical burden. Five RCTs or post hoc analysis of RCTs contributed data on clinical burden. Of these five studies, a single RCT provided clinical burden data of clinically relevant elevated Lp(a) thresholds (≥50 mg/dL) [35] and the remaining four RCTs or post hoc analysis defined elevated Lp(a) as ≥59.6 mg/dl [47], >73.7 mg/dL [48], ≥29.4 nmol/L [49] and >165 nmol/L [50]. The assessment of five RCTs or post hoc analysis of RCTs revealed heterogeneity in terms of patient population (i.e., overall ASCVD population vs the subgroup of ASCVD population with specific LDL-C and HDL-C levels and on specific treatments) [47]; elevated Lp(a) and reference or low Lp(a) thresholds; comorbidities; biomarkers, including HDL-C, LDL-C, TC, HbA1C, and hsCRP; gender distribution; and risk factors for ASCVD (smoking history, history of CAD, MI, stroke, and PAD events) [35,48–50]. MACE was the only outcome that was commonly reported across the trials. However, the definition of MACE varied widely between the five studies, hence, pooling of results for this outcome was not feasible.

Of the 56 observational studies, only the studies that reported clinically relevant elevated Lp(a) thresholds were considered for meta-analysis. Twelve of the 56 studies reporting clinical burden for elevated Lp(a) levels defined as ≥30 mg/dL were analyzed for the feasibility of meta-analysis. Of these 12 studies, seven were ruled out for meta-analysis due to variability in patient population and reference Lp(a) thresholds [20,21,24,25,27,31,32]. Six studies were excluded for meta-analysis due to variability in definition of outcomes, including stroke [28,30,43], MI [19,28,30,43], revascularization [28,30,43], and MACE [20,22,28,30,43]. Finally, three studies reporting CV mortality for patients with elevated Lp(a) levels ≥30 mg/dL (vs <30 mg/dL) were ruled out for meta-analysis as one study did not provide the sample size for Lp(a) categories as well as baseline characteristics and the two remaining studies were heterogeneous for patient characteristics [22,28,30].

Five of 56 studies reporting clinical burden for elevated Lp(a) levels defined as ≥50 mg/dL were evaluated for the feasibility of meta-analysis. The assessment revealed heterogeneity in the patient population [51]; reference or low Lp(a) thresholds; comorbidities; biomarkers; gender distribution, and risk factors for ASCVD [32–34,41]. For these observational studies, as with RCTs and post hoc analysis, the results for MACE (the only commonly reported outcome) could not be pooled due to varying definitions.

One study, each reported clinical burden for ≥70 mg/dL [36] and ≥75 nmol/L [37]. Two studies reported clinical burden for ≥150 nmol/L [14,40]; ≥100 nmol/L [38,40]; ≥125 nmol/L [39,40], and ≥200 nmol/L [41,42], each. These studies were ruled out for meta-analysis due to limited evidence. No study reported the clinical burden for Lp(a) levels ≥90 mg/dL, ≥180 mg/dL, and ≥300 mg/dL or ≥90 nmol/L, ≥400 nmol/L, and ≥430 nmol/L. The reasons for exclusion of the observational studies for meta-analysis are listed in Table 3.

**Table 3:** Reasons for exclusion of observational studies for meta-analysis.

Lp(a) threshold	MACE	MI	Stroke	Revascularization	CV mortality
≥30 mg/dL	N = 11 • Variability in reference Lp(a) thresholds • Variability in outcome definitions •Variability in population	N = 6 • Variability in reference Lp(a) thresholds • Variability in outcome definitions • Variability in population	N = 5 • Variability in reference Lp(a) thresholds • Variability in outcome definitions • Variability in population	N = 6 • Variability in outcome definitions - Variability in population	N = 6 • Variability in reference Lp(a) thresholds • Variability in population • Nonavailability of sample size • Variability in baseline characteristics
≥50 mg/dL	N = 3 • Variability in reference Lp(a) thresholds • Variability in outcome definitions	N = 1 • Scarcity of data	N = 1 • Scarcity of data	N = 1 • Scarcity of data	-
≥70 mg/dL	N = 1 • Scarcity of data	N = 1 • Scarcity of data	-	N = 1 • Scarcity of data	-
≥90 mg/dL	-	-	-	-	-
≥180 mg/dL	-	-	-	-	-
≥90 nmol/L	-	-	-	-	-
≥75 nmol/L	N = 1 • Scarcity of data	-	-	-	-
≥100 nmol/L	N = 1 • Scarcity of data	-	N = 2 • Scarcity of data	N = 1 • Scarcity of data	-
≥125 nmol/L	N = 2 • Scarcity of data	-	N = 1 • Scarcity of data	N = 1 • Scarcity of data	-
≥150 nmol/L	N = 1 • Scarcity of data	N = 1 - Scarcity of data	N = 2 • Scarcity of data	N = 2 • Scarcity of data	-
≥200 nmol/L	N = 2 • Scarcity of data	-	-	-	-
≥400 nmol/L	-	-	-	-	-
≥430 nmol/L	-	-	-	-	-

CV: Cardiovascular; Lp(a): Lipoprotein(a); MACE: Major adverse cardiac event; MI: Myocardial infarction.

### Quality of studies

The observational studies performed well on the NOS (average score: 6; range: 4 to 7 stars). Most of the studies scored ≥6 stars (n = 19, 73%) (S5 Table). Based on the Cochrane Collaboration’s Risk of Bias Tool for RCTs, the single included RCT was conducted well, and all the parameters had a low risk of bias (S6 Table).

## Discussion

Approximately 1.4 billion people worldwide (i.e., 20% of the global population) are living with elevated Lp(a) levels (≥50 mg/dL or ≥125 nmol/L) and are at high risk of developing ASCVD [7]. This SLR provides a holistic overview of current evidence on the clinical burden associated with clinically relevant elevated Lp(a) levels in patients with ASCVD in the secondary prevention setting. Moreover, the SLR showcases the available evidence for humanistic and economic burden of elevated Lp(a) levels in patients with ASCVD. The evidence shows significant association between elevated Lp(a) levels and an increased risk of MACE as well as revascularization. Most of the studies reporting CV mortality, all-cause mortality, MI, and stroke showed significant association or trend for association between elevated Lp(a) levels and the respective outcomes. The studies reporting humanistic burden reported higher cognitive impairment, increased disability related to stroke, and reduced quality of life in patients with elevated Lp(a) levels [44–46]. The single study included for economic burden reported inconclusive results for association between economic burden and elevated Lp(a) levels [43].

The most reported elevated Lp(a) threshold, defined as Lp(a) level ≥30 mg/dL (recommended by Chinese guidelines for the management of CV risk in adults with dyslipidemia [12] and EAS [11]), was found to be significantly associated with MACE [20–22,25,27,28,31,43] and revascularization [31,32,43]. For the remaining CV outcomes, including CV mortality, all-cause mortality, MI, and stroke, most of the included studies reported a trend for association between elevated Lp(a) levels and CV outcomes.

All except one study included for elevated Lp(a) levels defined as ≥50 mg/dL (as recommended by global WHO and IFCCLM guidelines; American NLA and AHA/ACC guidelines, and CCS guidelines [9]) reported significant association of elevated Lp(a) levels with MACE [32,34], MI [34], stroke [34], and revascularization [33]. The single RCT included for this threshold showed a trend for association between elevated Lp(a) (≥50 mg/dL) and an increased risk of MACE; however the association was not significant [35].

For elevated Lp(a) levels defined as ≥70 mg/dL or 150 nmol/L (recommended by the Australian integrated guidance for patients with FH with progressive clinical ASCVD [13]), most of the included studies reported a significant positive association of elevated Lp(a) levels with MACE [36,40], MI [36] and revascularization [14,36,40]. Of these studies, a single study reported data for ≥70 mg/dL [36], while two studies reported data for 150 nmol/L [14,40].

The single study that defined elevated Lp(a) levels as ≥75 nmol/L (recommended by EAS [11]) showed a significant association of elevated Lp(a) levels with MACE [37].

The two studies included for elevated Lp(a) levels defined as ≥100 nmol/L (recommended by WHO, IFCCLM, and NLA guidelines [9]) reported significant association between elevated Lp(a) levels and revascularization as well as stroke [40]; however, they showed a trend for association with MACE [39] and all-cause mortality [39,40].

The 2019 Heart UK guidelines defined Lp(a) levels ≥200 nmol/L as a high risk factor for CVD [9]. Both studies included for this threshold found a significant association of elevated Lp(a) levels with an increased risk of MACE [41,42].

Most of the studies reporting the association of clinical events with elevated Lp(a) levels showed a significant correlation and only about 30% of the studies did not show a significant association. Multiple factors could explain this variation, including differences in sample size, follow-up duration, baseline risk, and ethnicities. The follow-up duration of the included studies ranged from 1 year [30,38,45,46] to 11 years [14]. Interestingly, the average follow-up duration of the studies reporting significant association was approximately 4 years (n = 20, range: 1 to 11 years) [14,19–22,24–27,30–34,36,37,40–43], and for studies that did not report any association, it was 2.6 years (n = 8, range: 1 to 9 years) [24,28,30,35,38–40,43]. Hence, studies with shorter follow-up duration might be unable to establish the association of potential risk factors such as Lp(a) levels with mid-term and long-term outcomes. Most studies that showed significant associations with CV outcomes had higher sample size (an average of 3846 patients) [14,19–22,24–27,30–34,36,37,41–43], while most studies with smaller sample size (an average of 2032 patients) did not reach such conclusions [24,28,30,35,38,39,43]. A study by Welsh et al. conducted on a cohort of 413,734 individuals was not considered for sample size–related analysis as it was an outlier and could have created a bias [40].

Notably, the CV risk associated with Lp(a) remains irrespective of LDL-C levels [6]. The Copenhagen General Population Study reported that the incidence rate of MACE associated with elevated Lp(a) levels (>50 mg/mL) was maintained despite varying LDL-C levels [41]. In this SLR we identified a study that reported insights from the FOURIER trial focused on patients with CVD who were given the LDL-lowering therapy, evolocumab. The study reported that patients who achieved Lp(a) >29 nmol/L had significantly higher residual CV event rates at 3 years despite achieving LDL-C levels <70 mg/dL compared with patients who had achieved Lp(a) ≤ 29 nmol/L [50]. In corroboration, three prospective cohort studies (two from China [20,52] and one from North America [36]) reported significant association of elevated Lp(a) levels with an increased risk of MACE (HR [95% CI] range: 1.26 [1.07–1.48] to 1.59 [1.03–3.13]) and CV mortality (HR [95% CI]: 1.31 [1.08–1.6]) in patients with ASCVD with low LDL-C levels (vs low Lp(a) levels). Currently, therapies that target high LDL-C levels and lower the risk of ASCVD are available. However, there are no approved therapies that lower the Lp(a) levels and thereby lower the residual CV risk. Progressive CVD with Lp(a)-hyperlipoproteinemia has been approved as separate indication in the guidelines of statutory health insurance funds in Germany for apheresis [53]. Furthermore, Australian integrated guidance on enhancing care of patients with FH with ASCVD and elevated Lp(a) levels (≥150 nmol/L) has recommended apheresis to reduce ASCVD progression in patients who cannot achieve LDL-C targets despite maximally tolerated drug therapy [13]. Apheresis, an extracorporeal process of separating blood components, has been shown to reduce apolipoprotein-B–containing LDL-C and Lp(a). Moreover, it is hypothesized that the removal of blood factors. including fibrinogen, coagulation factors, thrombogenic factors, complement factors, inflammatory factors, and adhesion molecules, may reduce coagulation and improve endothelial function. Even though the efficacy of apheresis is unquestionable, apheresis might mediate its treatment effects through reductions in LDL-C or Lp(a) levels or by improvement in endothelium-dependent vasodilation [54,55]. Additionally, this invasive procedure is associated with several adverse events, including the risk of venipuncture complications, hypotensive episodes, and excessive bleeding [56]. Studies have shown that lowering Lp(a) by 50 mg/dL in the secondary prevention setting may reduce MACE risk by 22% over a short-term period and 45% over lifetime [41,57,58]. Thus, effective Lp(a)-targeting therapies for reducing the residual risk of secondary CV events remains an unmet need.

This SLR provides a systematic assessment of clinical burden associated with clinically relevant elevated Lp(a) levels in populations with secondary prevention of ASCVD. Furthermore, it also captures evidence for humanistic and economic burden of elevated Lp(a) levels in patients with ASCVD. This SLR is not restricted to any geography or study design and therefore provides a holistic overview of the burden of elevated Lp(a). Nonetheless, this SLR has some limitations, such as including only English-language studies. None of the included studies reported the association between Lp(a) levels and CV outcomes in different ethnic subpopulations. A study by Brandt et al. on a nationally representative cohort from the United States of America (USA) in the primary prevention setting revealed that Mexican-Americans with elevated (50 mg/dL) Lp(a) levels had the highest risk of MI compared with non-Hispanic Whites and non-Hispanic Blacks [59]. Another large case-control study in the primary prevention setting from the USA reported that the risk of stroke significantly increased with elevated Lp(a) levels in African Americans but not in Whites and Hispanics [60]. Given the ethnicity-driven impact of Lp(a) on CVD outcomes in the primary prevention setting, lack of similar studies in the secondary prevention setting is another gap identified through this SLR.

The clinical association of Lp(a) with outcomes did not vary much across geographies as exemplified by MACE and revascularization, which were found to be significantly associated with elevated Lp(a) for all the geographies. Studies reporting the influence of gender on the interaction between Lp(a) levels and CV outcomes were not available in the secondary prevention setting. However, a few studies in the primary prevention setting have reported that the association between elevated Lp(a) levels and the risk of MI or stroke is gender sensitive. For both outcomes, the risk of CV outcome increased with elevated Lp(a) levels in male but not in female patients [59,60]. Lp(a) levels have been shown to be responsive to estrogen levels, which wane in elderly women during and after menopause. However, the menopausal state of women was not reported in these studies [59]. Very few studies (N = 4) reported the subgroup data for different age groups. None of these studies could establish the impact of age on CV outcomes in patients with elevated Lp(a) levels [20,24,32,38]. These findings warrant further research to understand the underlying effects of gender and age on Lp(a) levels and associated CV risk in the secondary prevention setting.

Patients in the included studies had different ASCVD conditions and different comorbidities, which may confound the results if there is lack of adjustment [61]. The varied thresholds determining the exposed group (with elevated Lp(a) levels) and unexposed group (with low Lp(a) levels) in the included studies is a major limitation of this SLR. The skewness in Lp(a) thresholds might have implications on the sample size in exposed and unexposed groups. This would lead to the comparison between elevated and low Lp(a) populations with unmatched sample size. The variability in the methods for Lp(a) measurements (immunoturbidimetry, enzyme-linked immunosorbent assay, or immunonephelometry), time of Lp(a) measurement, and processing of samples may introduce confounders and enrich the underestimated or overestimated Lp(a) values across the subgroups [62]. Nearly three-fourths of the included studies in this SLR either reported crude estimates or estimates adjusted for common covariates, including age and gender. Few studies adjusted for comorbidities, smoking status, family history, lipid profile, and lipid-lowering medications. These limitations warrant appropriately designing real-world evidence studies by adopting adequate methodologies for statistical adjustments for critical covariates and definitions of elevated and low Lp(a) levels to illustrate the impact of this causal CV risk factor on ASCVD outcomes.

A major limitation of this SLR is that we synthesized and presented the findings qualitatively as a meta-analysis was not feasible for the included evidence due to heterogeneity in patient population; reference thresholds (or low Lp(a) levels); comorbidities; biomarkers; gender distribution, risk factors for ASCVD; and definition of outcomes. Finally, limited studies evaluating the economic and humanistic burden of elevated Lp(a) was another key gap identified from this SLR.

## Conclusions

The findings of this SLR indicate that patients with ASCVD with elevated Lp(a) are at an increased risk of MACE, CV mortality, MI, stroke, revascularization, cognitive impairment, disability, and compromised quality of life. The evidence suggests that Lp(a) is an important biomarker that should be tested as part of routine lipid screening and ASCVD risk profiling. Absence of approved treatments for the secondary prevention of ASCVD in patients with elevated Lp(a) levels mandates the ongoing clinical research to provide therapies that reduce the CV risk associated with elevated Lp(a) levels. Our SLR warrants further high-quality studies in different geographies and ethnic groups to characterize the implications of elevated Lp(a) on clinical burden. Moreover, new studies assessing the economic and humanistic burden of ASCVD are needed to fill the lacunae caused by the paucity of evidence in this domain.

## Supporting information

S1 Table PRISMA checklist. (DOCX)

S2 Table Embase and MEDLINE search strategy using Embase.com interface (searched on March 28, 2022). (DOCX)

S3 Table MEDLINE Epub ahead of print, in-process, and other nonindexed citations search strategy using PubMed.com interface (searched on March 28, 2022). (DOCX)

S4 Table Inclusion and exclusion criteria for systematic literature review. (DOCX)

S5 Table Newcastle-Ottawa risk of bias assessment of the included case-control and observational studies. (DOCX)

S6 Table Cochrane’s Collaboration Risk of Bias assessment of the included RCTs. (DOCX)
