# Supplementary material for: Burden of elevated lipoprotein(a) among patients with atherosclerotic cardiovascular disease: Evidence from a systematic literature review and feasibility assessment of meta-analysis
Source: PLoS One. 2023 Nov 20;18(11):e0294250. doi: 10.1371/journal.pone.0294250 (PMC10659166; doi:10.1371/journal.pone.0294250)
Supplement: S2 Table — (DOCX) [file pone.0294250.s002.docx]

**S2 Table. Embase and MEDLINE search strategy using Embase.com interface (searched on March 28, 2022)**

| **Parameter** | **S. No.** | **Search terms** |
| --- | --- | --- |
| **Lp(a)** | **1** | 'lipoprotein a'/syn OR 'lipoprotein a' |
|  | **2** | 'lipoprotein-little-a' OR 'l-p-little-a' OR 'l-p-a' OR 'lipoprotein(a)' OR 'lipoprotein(a-)' OR 'lp(a)' OR 'lipoprotein a' OR ‘lipoprotein (a)’ |
|  | **3** | 'apolipoprotein a'/de OR 'apolipoprotein a' |
|  | **4** | #1 OR #2 OR #3 |
| **Epidemiology** | **5** | 'epidemiology'/de OR 'incidence'/syn OR 'prevalence'/syn OR trend* OR epidemiol*:ab,ti OR prevalen*:ab,ti OR inciden*:ab,ti OR (((natural* OR disease*) NEAR/3 (progress* OR course* OR history*)):ab,ti) |
|  | **6** | 'high*':ab,ti OR 'increas*':ab,ti OR 'elevated':ab,ti OR 'rais*':ab,ti OR 'level*':ab,ti OR 'distribution*':ab,ti OR 'proportion*':ab,ti |
|  | **7** | #5 OR #6 |
| **Screening** | **8** | 'screen*':ab,ti OR 'diagnos?s':ab,ti OR 'examination':ab,ti OR 'detect*':ab,ti OR  'measurement':ab,ti OR 'value*':ab,ti |
| **Disease management** | **9** | 'disease management'/de |
|  | **10** | 'practice guideline'/syn OR 'consensus development'/syn OR ((treat* OR manage* OR therap*) NEAR/5 (guideline* OR practic* OR recommend* OR pattern* OR algorithm)) |
|  | **11** | 'therapy patter*' OR 'treatment patter*' OR 'treatment strateg*' OR 'treatment regimen*' OR 'standard treatment' OR 'standard of care' OR soc OR switch* OR substitution OR discontin* OR dropout* OR 'drop-out*' OR restart* OR combination OR augment* OR pattern* OR 'add-on*' OR addon* OR adjuvant* |
|  | **12** | ('cholesterol lowering' OR 'hypocholesterolemic' OR 'ldl-c lowering' OR 'ldl c lowering') AND (agent OR drug OR therapy OR treatment) OR ('hypocholesterolemic agent'/syn) |
|  | **13** | #9 OR #10 OR #11 OR #12 |
| **Burden** | **14** | ('years' NEAR/3 'lost') OR 'daly' OR 'disability adjusted life year' OR 'qol' OR 'quality of life' OR 'quality-of-life' OR 'disab*' OR 'quality adjusted life year' OR 'qaly' OR ('burden' NEAR/5 ('economic' OR 'societ*' OR 'caregiver' OR 'humanistic')) OR 'econom*' OR 'cost*' OR 'expen*' OR (('work' OR 'productiv*') NEXT/3 ('present*' OR 'absen*' OR 'los*')) OR 'resource utili*' OR 'healthcare utili*' OR (('patient' OR 'physician') NEXT/3 ('satisf*' OR 'preference' OR 'perspective')) OR 'hospitali*' OR 'length of stay' OR 'los' OR ('emergency')  NEXT/2 ('department' OR 'room')) OR 'hui' OR 'utilit*' OR 'health year equivalent' OR 'hye' OR 'adl' OR ('activit*' NEAR/2 'daily' NEAR/2 'living') OR 'comorbidity'/exp OR 'morbidity'/exp OR 'mortality'/exp OR 'death':ab,ti OR 'survival':ab,ti OR 'die*':ab,ti OR 'major adverse cardiovascular event':ab,ti OR 'mace':ab,ti OR (('cardiovascular' OR 'cv') NEAR/2 'death*') OR myocardial infarction/syn OR 'non-fatal mi' OR 'nonfatal mi' OR 'non-fatal myocardial infarction' OR 'nonfatal myocardial infarction' OR 'non-fatal heart infarction' OR 'nonfatal heart infarction' OR 'brain ischemia'/exp OR 'heart muscle revascularization'/exp OR revascularization OR surgery OR procedure OR ‘visit*’ |
| **Lp(a) – Epi** | **15** | #4 AND #7 |
| **Lp(a) – Screening** | **16** | #4 AND #8 |
| **Lp(a) – Disease management** | **17** | #4 AND #13 |
| **Lp(a) – Burden** | **18** | #4 AND #14 |
|  | **19** | #15 OR #16 OR #17 OR #18 |
| **Initial hits** | **20** | #19 AND [2010-2022]/py |
| **Limits** | **21** | #20 AND ('editorial'/it OR 'letter'/it OR 'note'/it OR 'commentary'/it OR 'case report'/it OR 'case study'/it OR 'case series'/it) |
|  | **22** | #20 AND ('conference abstract'/it OR 'conference paper'/it OR 'conference review'/it) |
|  | **23** | #20 AND ([animal cell]/lim OR [animal experiment]/lim OR [animal model]/lim OR [animal tissue]/lim) NOT 'human'/de |
|  | **24** | #21 OR #22 OR #23 |
|  | **25** | #20 NOT #24 |
|  | **26** | #25 AND [english]/lim |
|  | **27** | #26 AND ([cochrane review]/lim OR [systematic review]/lim OR [meta analysis]/lim) |
|  | **28** | #26 AND 'review'/it |
|  | **29** | #28 NOT #27 |
| **Final hits** | **30** | **#26 NOT #29** |
