# Supplementary material for: Burden of elevated lipoprotein(a) among patients with atherosclerotic cardiovascular disease: Evidence from a systematic literature review and feasibility assessment of meta-analysis
Source: PLoS One. 2023 Nov 20;18(11):e0294250. doi: 10.1371/journal.pone.0294250 (PMC10659166; doi:10.1371/journal.pone.0294250)
Supplement: S3 Table — (DOCX) [file pone.0294250.s003.docx]

**S3 Table. MEDLINE Epub ahead of print, in-process, and other nonindexed citations search strategy using PubMed.com interface (searched on March 28, 2022)**

| **Parameter** | **S. No.** | **Search terms** |
| --- | --- | --- |
| **Lp(a)** | **1** | (lipoprotein a) OR (lipoprotein (a)) OR (lipoprotein(a)) |
|  | **2** | apolipoprotein a |
|  | **3** | #1 OR #2 |
| **Epidemiology** | **4** | epidemiology OR incidence OR prevalence OR trend* |
|  | **5** | high* OR increas* OR elevated OR rais* OR level* OR distribution* OR proportion* |
|  | **6** | #4 OR #5 |
| **Screening** | **7** | screen* OR diagnos* OR examination OR detect* OR measurement OR value* |
| **Disease management** | **8** | disease management |
|  | **9** | (practice guideline) OR (consensus development) OR ((treat* OR manage* OR therap*) NEAR/5 (guideline* OR practic* OR recommend* OR pattern* OR algorithm)) |
|  | **10** | therapy patter* OR treatment patter* OR treatment strateg* OR treatment regimen* OR standard treatment OR standard of care OR soc OR switch* OR substitution OR discontin* OR dropout* OR 'drop-out* OR restart* OR combination OR augment* OR pattern* OR addon* OR addon* OR adjuvant* |
|  | **11** | hypocholesterolemic agent |
|  | **12** | (cholesterol lowering OR hypocholesterolemic OR ldl-c lowering)  OR ldl c lowering) AND (agent OR drug OR therapy OR treatment) |
|  | **13** | #8 OR #9 OR #10 OR #11 OR #12 |
| **Burden** | **14** | ('years' NEAR/3 'lost') OR 'daly' OR 'disability adjusted life year' OR 'qol' OR 'quality of life' OR 'quality-of-life' OR 'quality adjusted life year' OR 'qaly' OR 'clinical burden' OR 'humanistic burden' OR 'economic burden' OR 'societ* burden' OR 'caregiver burden' OR 'econom*' OR 'cost*' OR 'expen*' OR (('work' OR 'productiv*') NEXT/3 ('present*' OR 'absen*' OR 'los*')) OR 'resource utili*' OR 'healthcare utili*' OR (('patient' OR 'physician') NEXT/3 ('preference' OR 'perspective')) OR 'hospitali*' OR 'length of stay' OR 'los' OR ('emergency' NEXT/2 ('department' OR 'room')) OR 'hui' OR 'utilit*' OR 'health year equivalent' OR 'hye' OR 'adl' OR ('activit*' NEAR/2 'daily' NEAR/2 'living') OR 'comorbidity' OR 'morbidity' OR 'mortality' OR 'death' OR 'survival' OR 'die*' OR 'major adverse cardiovascular event' OR 'mace' OR (('cardiovascular' OR 'cv') NEAR/2 'death*') OR 'myocardial infarction' OR 'mi' OR 'non-fatal mi' OR 'nonfatal mi' OR 'non-fatal myocardial infarction' OR 'nonfatal myocardial infarction' OR 'non-fatal heart infarction' OR 'nonfatal heart infarction' OR 'brain ischemia' OR 'heart muscle revascularization' OR revascularization OR surgery OR procedure OR 'visit*' |
| **Lp(a) – Epi** | **15** | #3 AND #6 |
| **Lp(a) – Screening** | **16** | #3 AND #7 |
| **Lp(a) – Disease management** | **17** | #3 AND #13 |
| **Lp(a) – Burden** | **18** | #3 AND #14 |
| **Initial hits** | **19** | #15 OR #16 OR #17 OR #18 |
| **Final hits** | **20** | #19 AND (pubstatusaheadofprint OR inprocess[sb]) |
