# Supplementary material for: Burden of elevated lipoprotein(a) among patients with atherosclerotic cardiovascular disease: Evidence from a systematic literature review and feasibility assessment of meta-analysis
Source: PLoS One. 2023 Nov 20;18(11):e0294250. doi: 10.1371/journal.pone.0294250 (PMC10659166; doi:10.1371/journal.pone.0294250)
Supplement: S4 Table — (DOCX) [file pone.0294250.s004.docx]

**S4 Table. Inclusion and exclusion criteria for systematic literature review**

| **Inclusion criteria** | **Exclusion criteria** |
| --- | --- |
| **Patient Population:**   - Studies with adult patients (≥18 years) with established atherosclerotic cardiovascular disease and Lp(a) measurement, which included patients with: - Prior acute coronary syndrome, myocardial infarction, stable or unstable angina - Prior stroke - History of peripheral artery disease - Prior revascularization procedures (e.g., percutaneous coronary intervention, coronary artery bypass graft) - Coronary artery disease - Coronary heart disease - Coronary artery angiography - Coronary aortic valve stenosis | **Patient Population:**   - Studies with patients for prevention of atherosclerotic cardiovascular disease - Studies with other population - Studies with no information on Lp(a) assessment |
| **Intervention:**   - None | **Intervention:**   - None |
| **Comparators:**   - None | **Comparators:**   - None |
| **Outcomes:**   - Study details: country, year of publication, study design, and objective - Information of Lp(a) measurement: testing, assay, unit - Population and baseline clinical characteristics - Prevalence, incidence, or distribution of Lp(a) - Burden associated with Lp(a): clinical, humanistic, economic | **Outcomes:**   - Studies not reporting relevant outcomes |
| **Study designs:**   - Observational studies such as cohort study/follow-up study, longitudinal study, cross-sectional study, prospective study, retrospective study, case-control study, population-based study, registry, survey - Meta-analyses - Randomized clinical trials were considered for epidemiology only if sample size was >1,000 patients | **Study designs:**   - *In vitro* - Pharmacokinetics/pharmacodynamics studies |
| **Species**   - Humans | **Species**   - Animals |
| **Language:**   - Papers published in English only | **Language:**   - Non-English |
| **Country:**   - No restriction | **Country:**   - None |
| **Search timeframe:**   - Database searches: January 1, 2010, to October 31, 2020 | **Search timeframe:**   - Studies published prior to year 2010 |
| **Publication type:**   - Peer-reviewed full-text journal articles | **Publication type:**   - Reviews - Systematic reviews - Conference abstracts - Editorials, letters, editorials, commentaries - Case series, case reports |
