# Supplementary material for: Burden of elevated lipoprotein(a) among patients with atherosclerotic cardiovascular disease: Evidence from a systematic literature review and feasibility assessment of meta-analysis
Source: PLoS One. 2023 Nov 20;18(11):e0294250. doi: 10.1371/journal.pone.0294250 (PMC10659166; doi:10.1371/journal.pone.0294250)
Supplement: S5 Table — (DOCX) [file pone.0294250.s005.docx]

**S5 Table: Newcastle-Ottawa risk of bias assessment of the included case-control and observational studies**

| **Study** | **Representativeness of the exposed cohort** | **Selection of the non exposed cohort** | **Ascertainment of exposure** | **Demonstration that outcome of interest was not present at start of study** | **Comparability of cohorts on the basis of the design or analysis** | **Assessment of outcome** | **Was follow-up long enough for outcomes to occur** | **Adequacy of follow up of cohorts** | **Total** |
| --- | --- | --- | --- | --- | --- | --- | --- | --- | --- |
| Galasso 2021 | 1 | 0 | 1 | 0 | 1 | 1 | 1 | 1 | 7 |
| Gao 2021 | 1 | 0 | 1 | 0 | 1 | 1 | 1 | 1 | 6 |
| Liu 2021 | 1 | 0 | 1 | 1 | 1 | 1 | 1 | 1 | 7 |
| Sang 2021 | 1 | 0 | 1 | 1 | 1 | 1 | 1 | 1 | 7 |
| Wu 2021 | 1 | 0 | 1 | 0 | 1 | 1 | 0 | 0 | 4 |
| Golledge 2020 | 1 | 0 | 1 | 1 | 0 | 1 | 1 | 1 | 6 |
| Liu 2020b | 1 | 0 | 1 | 1 | 0 | 1 | 1 | 1 | 6 |
| Zhang M 2020 | 1 | 0 | 1 | 1 | 1 | 1 | 1 | 1 | 7 |
| Zhang 2020b | 1 | 0 | 1 | 1 | 1 | 1 | 1 | 0 | 6 |
| Zhu 2021 | 1 | 0 | 1 | 1 | 0 | 1 | 1 | 1 | 6 |
| Cai 2019 | 1 | 0 | 1 | 1 | 0 | 1 | 0 | 0 | 4 |
| Nicholls 2010 | 1 | 0 | 1 | 1 | 0 | 1 | 1 | 1 | 6 |
| Yoon 2021 | 1 | 0 | 1 | 0 | 1 | 1 | 1 | 1 | 6 |
| Bigazzi 2021 | 1 | 0 | 1 | 0 | 1 | 1 | 1 | 1 | 6 |
| Sanchez Muñoz-Torrero 2018 | 1 | 0 | 1 | 1 | 0 | 0 | 1 | 0 | 4 |
| Wong 2021 | 1 | 0 | 1 | 0 | 1 | 1 | 1 | 1 | 6 |
| Wang 2020 | 1 | 0 | 1 | 1 | 0 | 1 | 1 | 1 | 6 |
| Arnold 2021^∫^ | 1 | 0 | 1 | 1 | 1 | 1 | 1 | 1 | 7 |
| Wohlfahrt 2021 | 1 | 0 | 1 | 0 | 1 | 1 | 1 | 0 | 5 |
| Gencer 2019 | 1 | 0 | 1 | 1 | 0 | 1 | 0 | 1 | 5 |
| Patel 2020 | 1 | 0 | 1 | 1 | 0 | 1 | 0 | 0 | 4 |
| Welsh 2020 | 1 | 0 | 1 | 1 | 0 | 1 | 1 | 0 | 5 |
| Madsen 2020 | 1 | 0 | 1 | 1 | 0 | 1 | 1 | 1 | 6 |
| Waissi 2020 | 1 | 0 | 1 | 1 | 0 | 1 | 1 | 1 | 6 |
| Yang 2022 | 1 | 0 | 1 | 0 | 1 | 1 | 1 | 1 | 6 |
| Bao 2021 | 1 | 0 | 1 | 0 | 1 | 1 | 0 | 0 | 4 |
| Jiang 2021 | 1 | 0 | 1 | 0 | 1 | 1 | 1 | 0 | 5 |
| Li 2021 | 1 | 0 | 1 | 1 | 1 | 1 | 1 | 1 | 7 |
