# Supplementary material for: Burden of elevated lipoprotein(a) among patients with atherosclerotic cardiovascular disease: Evidence from a systematic literature review and feasibility assessment of meta-analysis
Source: PLoS One. 2023 Nov 20;18(11):e0294250. doi: 10.1371/journal.pone.0294250 (PMC10659166; doi:10.1371/journal.pone.0294250)
Supplement: S6 Table — (DOCX) [file pone.0294250.s006.docx]

S6 Table. Cochrane’s Collaboration Risk of Bias assessment of included RCTs

| **Study** | **Allocation concealment** | **Was knowledge of the allocated intervention adequately prevented during the study?** | **Were outcome assessment was performed blindly?** | **Were incomplete outcome data adequately addressed?** | **Are reports of the study free of suggestion of selective outcome reporting?** | **Was the study apparently free of other problems that could put it at a high risk of bias?** |
| --- | --- | --- | --- | --- | --- | --- |
| Puri 2017 | Low risk | Low risk | Low risk | Low risk | Low risk | Low risk |
